# Age Differences in Electronic Mental Health Literacy: Qualitative Study

**DOI:** 10.2196/59131

**Published:** 2024-07-16

**Authors:** Richard Huan Xu, Lidan Tian, Liling Zhu, Yuan Cao, Sherry Kit-wa Chan, Dong Dong, Wai-ling Annie Cheung, Eliza Lai-yi Wong

**Affiliations:** 1 Department of Rehabilitaion Sciences Faculty of Health and Social Sciences Hong Kong Polytechnic University Kowloon China (Hong Kong); 2 Department of Applied Social Science Faculty of Health and Social Sciences Hong Kong Polytechnic University Kowloon China (Hong Kong); 3 Department of Social Work and Social Administration The University of Hong Kong Hong Kong China (Hong Kong); 4 Department of Psychiatry The University of Hong Kong Hong Kong China (Hong Kong); 5 JC School of Public Health and Primary Care The Chinese University of Hong Kong Shatin China (Hong Kong)

**Keywords:** eHealth literacy, mental health, mental health literacy, age-related difference, electronic mental health literacy, eMHL

## Abstract

**Background:**

Electronic mental health literacy (eMHL) is critical for accessing and effectively using digital mental health resources. However, there is a paucity of research on how eMHL varies across age groups.

**Objective:**

This study aimed to investigate differences in eMHL among young, middle-aged, and older adults; provide insights into the needs, behaviors, and attitudes of different age groups in relation to digital mental health resources; and ultimately, inform the improvement of mental health services.

**Methods:**

A qualitative investigation was conducted to examine the differences in eMHL across different age demographics in the Chinese population in 2023. The study sample comprised 3 distinct age groups: 18-34 years, 35-64 years, and 65 years and older. Participants were recruited through purposive sampling to ensure a diverse representation of the population. Data were collected through semistructured one-on-one interviews, which allowed for in-depth exploration of individual experiences and perceptions. The gathered data were subsequently subjected to rigorous thematic analysis to enable the identification and interpretation of recurring patterns and themes.

**Results:**

The principal outcomes derived from these interviews were synthesized into 5 distinct dimensions: emotional needs, use of digital mental health resources, assessment of digital mental health information, engagement with social media to regulate emotions, and coping strategies. These dimensions were uniformly observed across the 3 age groups.

**Conclusions:**

We identified differences in knowledge, skills, and attitudes regarding the use of web-based information for managing mental health problems between the 3 age groups. The findings highlight the importance of age-specific strategies for improving eMHL.

## Introduction

Traditional mental health services frequently operate at their maximum capacity due to constrained resources. Consequently, some individuals are deprived of the care that they require. Digital mental health platforms, encompassing both mobile and web-based systems, are a potential solution to this challenge [1]. These platforms, which include services such as digital counseling, forums, and chat groups, offer an alternative avenue for individuals who are reluctant to engage in face-to-face therapy or feel alienated by traditional services [2]. Studies have reported age differences in the use of digital mental health platforms. For example, a study in Ireland reported that about half of the respondents aged 18 to 25 years used social media and apps for mental health support [3]. Another study in the United Kingdom showed that nearly two-thirds of young people with a history of mental health needs had accessed digital mental health tools [4]. In contrast, a study in the United States found that only 14.2% of middle-aged individuals used digital mental health tools to cope with stress related to the COVID-19 pandemic [5]. Older adults exhibit a lower acceptance of digital mental health resources than middle-aged and younger adults [6]. This could be due to their general lack of digital skills and discomfort with new technologies or because digital interventions are typically designed for advanced users [7-9].

Research has found that age differences in the use of the internet to manage mental health can be attributed to various factors. The health information demands of older persons differ from those of younger adults [10], including those regarding chronic diseases, social isolation [11], and physical and cognitive limitations due to the natural aging process [12,13]. Studies have also highlighted generational differences in information-seeking behavior in nonhealth contexts. For children and young people, moderate internet use can have a small positive impact on their well-being. Young people use the internet to manage emotional problems, although this use could become a maladaptive coping strategy. They may use the internet to distract themselves from negative emotions; escape from reality; or regulate feelings of loneliness, depression, or anxiety [14]. However, social media platforms can exacerbate feelings of anxiety, isolation, and depression, especially among young people who spend time on the internet at the expense of in-person interaction [15,16]. In summary, younger and older adults require different approaches to health education, treatment options, and digital support systems to effectively address their unique challenges and promote their overall well-being.

Electronic mental health literacy (eMHL) is an emerging concept that extends mental health literacy into the digital era. It reflects the ability of individuals to access, understand, and use digital mental health information and services [17], an ability that is essential for the recognition, management, and prevention of mental health issues.

Studies on eMHL have primarily focused on younger populations. For instance, Cormier et al [18] found a close relationship between low eMHL and an increased risk of mental health problems. Another study on young adults found that those with low eMHL are at high risk of experiencing a mental health disorder [19]. However, mental health issues affect individuals across all age groups, from children and adolescents to adults and older individuals. It is crucial to understand the prevalence and impact of these issues across different age brackets to develop effective treatment strategies. The internet plays an increasingly important role in understanding and addressing the mental health problems of individuals across various age groups. It offers a wealth of resources and information and facilitates access to communities for mental health support. Currently, there is a lack of qualitative evidence on the eMHL of people across different age groups. Research on this issue is necessary to understand how different age groups perceive and use eHealth resources. This knowledge could inform the design and implementation of more effective and personalized digital health interventions. Furthermore, it could aid in the development of strategies to enhance digital health literacy, thereby improving health outcomes for individuals across all age brackets. Therefore, this study investigated age differences in eMHL between young, middle-aged, and older adults. The findings offer insights into the needs, behaviors, and attitudes of individuals of different age groups in relation to digital health, thus informing efforts to improve mental health services.

## Methods

### Design and Sampling

We conducted a qualitative study to understand individuals’ experiences of seeking and using information on the internet to manage their mental health. The study involved in-depth, semistructured interviews with participants from the 3 adult age groups suggested in the Hong Kong Census guidelines: young adults (18-34 years), middle-aged adults (35-64 years), and older adults (65 years and older). The study was conducted from May to September 2023. Participants were recruited via research flyers, which were shared on university notice boards and distributed via the university’s electronic mass mail system to all university visitors, students, staff, collaborators, and alumni. The flyers included a detailed study description, participation criteria, information about the offline interviews (each approximately 60 minutes long), the impact of the study, and the researchers’ contact information. Interested individuals filled out a web-based questionnaire in which they provided their background information. The research team then screened the prospective participants based on age, gender, and their experiences with counseling. Participants had to fulfill the following criteria: (1) be citizens and residents of Hong Kong, (2) be Cantonese speakers, (3) be aged between 18 and 70 years, and (4) have experienced mild to severe mental health problems (as determined by the Depression, Anxiety and Stress Scale-21 Item [DASS-21] questionnaire [20]).

### Data Collection Procedure

As there is no gold standard for sample sizes in qualitative studies, we followed Creswell’s [21] recommendation and aimed to recruit 6-8 participants from each age group, totaling 18-24 participants across the 3 groups. However, the final sample size was determined based on data saturation, the point at which no new themes emerged from the participants’ experiences. In total, 292 individuals registered for the survey by scanning the QR code on the flyers. Following the screening of participants’ mental health status, we identified 109 individuals with mild or severe mental health issues, as measured by DASS-21, as suitable for inclusion in the study. We then divided these eligible participants into 3 age groups. Each of these individuals was allocated a random number from 1 to 109. A research assistant then contacted them in ascending order of their numbers to organize interviews with them. Once their willingness to be interviewed in person and availability were confirmed, they were invited for the interview session in a university conference room. We stopped this process when saturation was reached, after which no further participants were interviewed.

Before the interview, the participants were given a consent form to review. Any doubts that they had were clarified by the research team. The form covered permissions for recording the interview, assurances of anonymity, and data retention periods. The semistructured interview was conducted after they had signed the informed consent form. In the interview, topics, such as the participants’ background, daily mental health experiences, and their knowledge about and interaction with digital mental health resources, were discussed. An interviewer experienced in qualitative research and fluent in Cantonese led the sessions with the help of a research assistant who took notes to capture important information.

### Ethical Considerations

The Research Ethics Committee at the Hong Kong Polytechnic University approved the study protocol and the procedure for obtaining informed consent (HSEARS20211130001). All of the participants provided written informed consent. The data generated in the study were anonymized. Each participant received a HK $100 (equal to US $12.8) local supermarket coupon after the interview as a token of appreciation.

### Data Analysis

The interview recordings were automatically transcribed using digital software (iFLYREC) and manually proofread. Thematic analyses were used for data synthesis to understand the participants’ perspectives on digital mental health resources and the approach they adopted when seeking professional help. We conducted a reflexive thematic analysis using a realist framework and integrated the inductive and deductive approaches. The analysis comprised six steps [22]: (1) data familiarization, (2) coding, (3) initial theme generation, (4) theme review, (5) theme definition or naming, and (6) writing of results. The 2 researchers (RHX and LZ) were responsible for familiarizing themselves with the data and generating codes and initial themes. This process involved line-by-line open coding and discussions between the 2 researchers for the first 3 transcripts (1 for each age group). Afterward, 2 researchers (RHX and LZ) reviewed and revised the codes and themes through focused coding of the first 3 transcripts and 3 additional transcripts (1 for each age group). Interviews were conducted in a preset order: 2 from the young adult group, followed by 2 from the middle-aged adult group, and then 2 from the older adult group, continuing in this sequence until the end. After each interview round, the authors reviewed the data for emerging themes or information. We checked for patterns, concepts, and categories. If new or relevant themes were discovered, we continued the interviews until they started yielding redundant information, indicating data saturation. To confirm saturation, we conducted 1 or 2 additional interviews in each age group. If these offered no new insights, we concluded that saturation was achieved and data collection was ended.

Thematic analysis was conducted in a stepwise manner. First, data related to the different age groups were analyzed individually. This required a thorough exploration of the data from each group to identify unique patterns and trends. The analysis began with data from the young adult group, followed by the middle-aged adult group, and finally, the older adult group. After the initial analysis, the findings for the different age groups were then synthesized. The synthesis allowed for the generation of overarching themes and patterns that spanned the age groups, providing a comprehensive understanding of the data as a whole. This process was conducted in a recursive and reflexive manner. All of the data were analyzed in Chinese, the language in which the interviews were conducted, to ensure accuracy. For the preparation of this paper, key findings and selected quotes were translated into English with the help of a translator from a professional translation company. The translator was a native English speaker with a good understanding of Chinese. All of the translations were jointly confirmed by the research team and the translator. Dedoose was used for coding and thematic analysis.

## Results

### Overview

**Figure 1:**
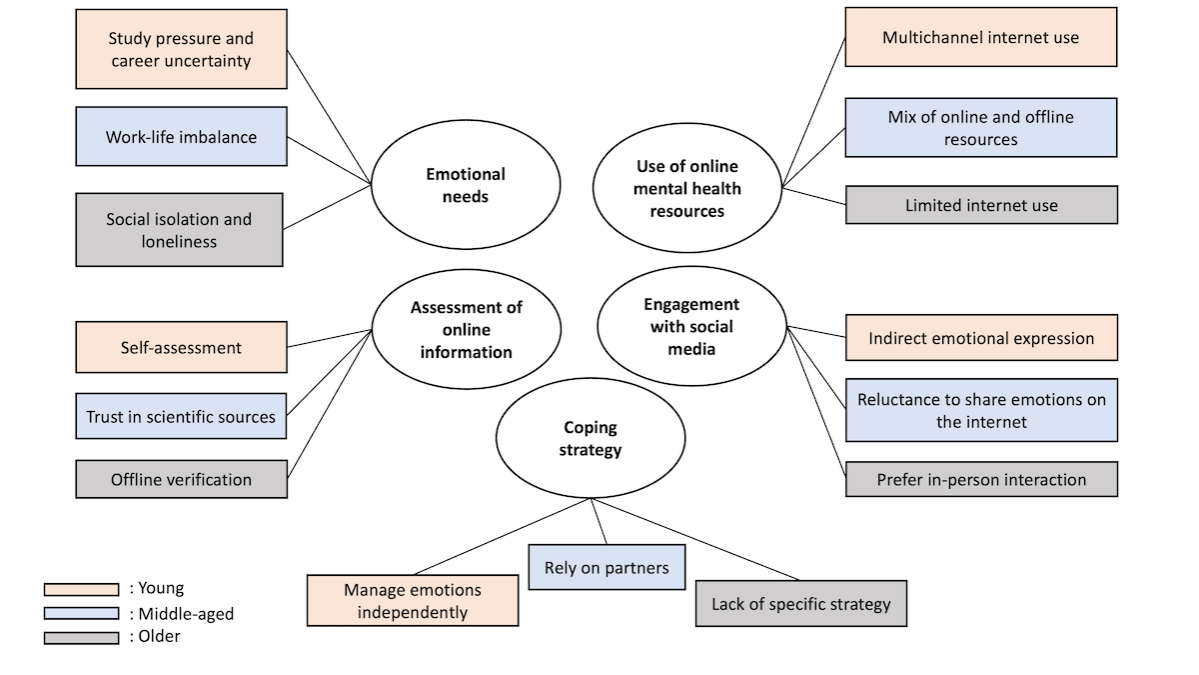
Thematic map of the findings.

We interviewed 30 participants across the 3 age groups, at which point our data reached saturation. Table 1 summarizes the backgrounds of the participants. The primary findings were synthesized into a thematic map (Figure 1). We organized them into 5 categories: emotional needs, use of digital mental health resources, assessment of digital mental health information, engagement with social media for emotional management, and coping strategies. These categories were applicable to all 3 age groups. In the selected quotes presented in this paper, the numbers referring to the participants are prefixed with Y, M, or O to indicate whether they belonged to the young adult, middle-aged adult, or older adult group, respectively.

**Table 1:** Demographics of participants of in-depth interview (N=30).

Characteristics				Values, n (%)
**Sex**				
	Male			14 (47)
	Female			16 (53)
**Age groups (years)**				
	18-34			10 (33)
	35-64			10 (33)
	≥65			10 (33)
**Marital status**				
	Unmarried			13 (43)
	Married			16 (53)
	Divorced or separated or widowed			1 (3)
**Employment**				
	Full-time employed			18 (60)
	Retired			5 (17)
	Student			3 (10)
	Part-time employed			1 (0.3)
	Unemployed			1 (0.3)
	Housewife			1 (0.3)
	Others			1 (0.3)
**Government assistance received**				
	No			30 (100)
**Educational level**				
	Secondary or below			3 (10)
	Postsecondary			5 (17)
	Tertiary or above			22 (73)
**Have seen a psychiatrist or received psychological counseling in the past year**				
	Yes			7 (23)
	No			23 (77)
**DASS-21^a^**				
	**Depression subscale**			
		Mild	1 (3)	
		Moderate	17 (57)	
		Severe	3 (10)	
		Extremely severe	9 (30)	
	**Anxiety subscale**			
		Mild	1 (3)	
		Moderate	9 (30)	
		Severe	7 (23)	
		Extremely severe	13 (43)	
	**Stress subscale**			
		Mild	7 (23)	
		Moderate	10 (33)	
		Severe	6 (21)	
		Extremely severe	7 (23)	

^a^DASS-21: Depression, Anxiety and Stress Scale-21 Item.

### Impact of Emotional Needs on Web-Based Behavior

#### Study Pressure and Career Uncertainty Among the Young Adults

The pressure of academia and entry into the workforce motivated the young participants to seek digital sources to deal with their emotions. A common theme among this group was uncertainty about education and job prospects, especially for those seeking internships or jobs. Some also mentioned that relationships caused them anxiety and depression.

I experienced a lot of stress while job hunting after graduation, so I often browsed online forums and videos for tips and motivation.

Y 012

#### Work-Life Imbalance Among the Middle-Aged Individuals

The middle-aged participants experienced noticeable anxiety and depression. A significant majority of them were employed and faced the delicate task of balancing work commitments with family responsibilities, driving their need to find flexible web-based support.

Given my demanding workload, I really need some easily accessible online resources that I can use whenever I have time, such as mental health chatbots.

M 013

Because I often feel anxious about my work, I go online to look for articles and resources on how to relieve my emotions.

M 026

#### Social Isolation and Loneliness in the Older Adults

Navigating life after retirement posed significant challenges for the older adult participants. The older adults found it crucial to address how to structure their daily lives following retirement to maintain their mental well-being. This necessity drove some to seek support online and offline.

After retirement and my father’s passing, I lived alone and felt extremely lonely and bored. I realised how isolating it could be for someone unable to go out and interact with others. I then sought help from social workers and a psychiatrist. I was also recommended to go online to find local community groups and activities to join.

O 010

### Use of Digital Mental Health Resources

#### Multichannel Internet Use Among the Young Adults

The young individuals, being adept at using the internet, leveraged various digital platforms for emotional relief. They engaged with autonomous sensory meridian response videos, virtual city tours, and resources providing insights into professional life, gaming, music, and short videos. While not directly addressing emotions, these activities provided effective relief in times of emotional difficulty. These participants also showed interest in mental health services for relaxation and personal growth.

I look for resources on many social media platforms, such as official accounts on the Little Red Book, Bilibili, and WeChat. I follow those that I resonate with.

Y012

I try watching motivational videos on YouTube to see if they help with my emotions.

Y 016

#### Use of a Mix of Online and Offline Resources Among the Middle-Aged Individuals

The middle-aged participants used a mix of traditional and new resources for emotional well-being. The digital resources that they used included platforms, such as YouTube and Facebook; popular science channels; and mental health services, such as “Shall We Talk” and “Know Yourself.” They sought professional help on the internet when needed and also explored indirect channels, such as religion and travel to self-manage their emotional health.

I learn emotional coping skills by watching instructional videos on YouTube, and I also pay attention to some innovative counselling services like Shall We Talk or Know Yourself.

M 013

I think some YouTube shorts on releasing stress or Facebook groups for anxiety issues could be helpful. But for serious problems I’d still need guidance from an online psychiatrist.

M 030

#### Restricted Internet Use Among the Older Adults

The older adults struggled with adopting digital resources because of unfamiliarity with the internet and the fear of scams, which are prevalent in Hong Kong. They searched for web-based information but preferred offline engagement with activities, such as lectures and social services. They were unfamiliar with platforms, such as Zoom (Zoom Video Communications) and ChatGPT (OpenAI), and complex web-based processes were challenging to them, particularly those with lower digital literacy.

I feel that information on the internet is not easy to find. What I’ve found are just some Youtubers who call themselves therapists, but I don’t really trust them.

O 003

I sometimes use the internet to find a mental health seminar or activity nearby. But I always end up going there in-person to talk to the social workers and psychologists face-to-face.

O 007

### Assessment of Digital Information in Mental Health

#### Self-Assessment by the Young Adults

The younger generation prioritized resources that helped them with their mental health issues, rather than focusing on their reliability. They trusted their own judgment over professional advice, as the latter often did not align with their unique circumstances. However, they were open to digital mental health services due to their cost-effectiveness, convenience, and potential to complement offline care.

If emotional support services are provided by non-governmental organisations themselves, their credibility is generally higher. But if it is provided by the government, I would have more reservations about using them.

Y 017

I have read posts on online forums or social media about mental health, but don’t fully trust them because none of them are actually verified as being accurate or inaccurate.

Y 018

#### Evidence-Based Assessment by the Middle-Aged Adults

The middle-aged individuals trusted science over government, actively engaged with digital content, and valued professional advice. They filtered the bulk of digital information using education and common sense, assessing reliability with a rational approach and considering factors, such as information quality, expert sources, and whether the information was shared by their peers.

When searching for mental health information online, I tend to trust scientific sources. However, there is too much contradictory information online, making it difficult to discern what is true or false. I also do not entirely trust professionals’ advice, I want to see the evidence they provide [to back their advice].

M 030

The middle-aged individuals often held conservative views on digital mental health services, believing that face-to-face interactions facilitate personal care and emotional connection in a manner that web-based platforms cannot. However, they acknowledged the value of digital services for emergencies or when schedules are too tight for in-person sessions. Notably, only a few of them were willing to invest in expensive web-based care.

Online services may allow some people to express themselves more boldly, because the counsellor only hears your voice and knows your name but doesn’t know what you look like.

M 007

During the COVID-19 pandemic, I was unable to go out. Online services were of some help during this exceptional time.

M 026

#### Offline Verification by the Older Adults

The older adults tended to prefer digital mental health services that were simple to navigate, trustworthy, and connected to offline nonprofit platforms. They often valued government, university, and nongovernmental organization services and trusted the firsthand experiences of others with mental health challenges.

I go online with a purpose. If there is some information that I need to check, I search for NGOs in places like Tsuen Wan or Kwai Chung, and then contact them for advice.

O 009

Web-based counseling, however, was not their preferred means of coping with mental health difficulties. Instead, they sought local resources—such as social workers—to alleviate their anxiety and depression. Those who had received a formal diagnosis often sought professional support from counselors or psychologists.

Not all online information is reliable, and online counselling only allows text responses. So I prefer to consult social workers or [mental health] professionals.

O 002

### Engagement With Social Media to Manage Emotions

#### Indirect Emotional Expression by the Young Adults

The young adults were the group that most frequently used social media to express their emotional needs to their digital social circles. They relied on social media and unique methods of expression to indirectly vent their emotions.

During my primary and secondary school years, I used to openly express my emotions online.

Y 012

Some of them found it immature to share unhappy feelings on social media. Others found solace in expressing those feelings on social media platforms in which they had fewer contacts, as if these platforms were a virtual tree hole (eg, a virtual tree hole in which one can safely reveal their difficult emotions). However, they typically reserved their main social media channels for sharing positive experiences.

I share some of my emotions on Facebook, as it is full of unknown people since I deleted some of my friends.

Y 017

I didn’t have many followers on Weibo. I used to post emotional things there.

Y 012

The interviews indicated, however, that as young people mature, they naturally gravitate toward their immediate social circles. Social media, in turn, becomes a space primarily for observing and connecting with moments in the lives of those in their social circle.

I have IG, Facebook, WeChat and so on, to see what my friends are posting, but I rarely post things myself.

Y 024

#### Reluctance to Share on the Internet Among the Middle-Aged Individuals

The middle-aged adult group exhibited a lack of trust in the internet due to concerns about privacy. They prioritized the security of their personal and family information on the internet. Because their negative emotions often stemmed from work or family issues, they typically found relief for those emotions offline. From their perspective, there was no need to share personal matters with their social circles on the internet, as doing so could lead to gossip.

I hardly share or repost anything online. I know there’s no need to act or voice my anger, even if I feel that something is unfair. I may wonder at the time, “Is this wrong?,” but I eventually let it go.

M 028

#### Preference for In-Person Interaction Among the Older Adults

The older adult group preferred to use the internet or mobile apps to connect with friends, but they also valued offline interactions when they were necessary. They tended to favor traditional phone calls over social media for communication. They typically did not share their emotions on the internet, as they perceived providing feedback via the internet to be complex.

If I want to talk to my classmates, we use a phone call. We don’t write things out in text online.

O 009

### Coping Strategies

There were no specific coping mechanisms that each group uniquely exhibited. Nevertheless, many of the interviewees, especially the young adults who needed time and space to heal from depression and anxiety, tried to manage their emotions independently. They did so to avoid receiving unsolicited advice from those who did not understand their perspective, as that often resulted in confusion and increased pressure.

I believe that my life should be under my own control, and what I post on social media is [in] my own space and I don’t care if others read it or not.

Y 020

There are times when I feel certain things cannot be said out loud. The more one tries to express them, the more annoyed people become. I feel that there needs to be a space for rational discussion, where everyone can truly open up and discuss freely.

Y 027

The middle-aged participants often relied on partners for emotional support, perhaps because their emotional challenges often came from work and family issues. The effectiveness of this support was dependent on the participants’ expectations of their confidant.

Sometimes I share with my wife, especially about matters regarding our kids. If something happens, like they throw a tantrum or cry, we may not know how to handle it. There may also be some minor conflicts when my wife and I have different opinions on family matters.

M 028

The interviews indicated, interestingly, that as individuals age, their inclination to share their emotional difficulties with friends or colleagues diminishes. This reluctance arises from a fear of burdening others with one’s emotional struggles.

As I age, I find it more difficult to express my emotions to those around me, because I worry that I’ll be a burden to them or that I’ll owe them favours I can’t repay.

O 001

I’ve seen some counsellors who just listen to you but don’t give advice. They just provide you with certain materials, and it is up to you to determine how to solve the problem on your own.

O 004

## Discussion

### Principal Findings

This study revealed significant variations in eMHL across different age groups. These disparities included differences in access to digital resources, assessment of digital information, and willingness to express emotions on the internet. The disparities highlight the need for multifaceted strategies for enhancing eMHL that consider generational preferences and trust levels [23,24]. We also discovered that individuals’ eMHL influences their behaviors and strategies for managing emotions. This finding underscores the need for and value of interventions to improve eMHL. The advent of digital technology presents increasing opportunities for digital mental health support and facilitates access to resources for mental health [25]. Our findings provide empirical evidence that individuals’ attitudes toward using information on mental health on the web and their skills in navigating the internet to find that information affect their access to mental health services. Therefore, it is essential to develop interventions that cater to the specific mental health needs and preferences of different age groups.

### Comparisons With Previous Studies

Our findings suggest that young people excel at gathering information from various sources and using multiple resources concurrently. This is consistent with the findings of Tirocchi et al [26], which highlight Generation Z’s (eg, those born between the mid-1990s and early 2010s) strong digital awareness and tendency to use several digital tools simultaneously, perhaps due to their extensive experience with the internet and their open-mindedness. Contrarily, middle-aged individuals often look for authoritative information and depend on their own judgment when seeking digital mental health resources. They generally favor a mix of traditional methods and new digital tools, valuing both professional advice and personal experience. Their approach may reflect a pragmatic and balanced method of seeking and evaluating information, including that on mental health. A study in China revealed that internet-based activities, such as reading news, watching videos, and playing games, effectively improved the mental health status of middle-aged individuals [27]. In contrast, older adults depend more on offline channels than digital resources, possibly due to their limited experience with and proficiency in using web-based platforms. Wei et al [28] found that older adults prefer face-to-face interactions and personal ties, which are better conducted through offline channels. Another study indicated that physical and cognitive impairments impede older people’s use of digital resources [29]. In summary, older adults are currently less able to fully leverage digital mental health resources. It is therefore essential to improve the eMHL of older adults to enable them to identify and use digital tools and training resources for managing their mental health. This would not only benefit the older adults themselves but also reduce the strain on caregivers and the overall health care system.

This study revealed that younger individuals often rely on their own judgment and the limited information available to them for decision-making. Influenced by social media and peer networks, they prioritize experiential and intuitive knowledge, especially in the use of digital information [30]. This could lead to a preference for personal experiences and opinions over traditional authoritative sources, for example, “making them susceptible to misinformation.” To tackle this challenge, promoting critical thinking and digital literacy is crucial. Numerous studies have emphasized how digital literacy impacts students’ ability to discern the veracity of digital information. For instance, a study on Generation Z students found that various aspects of digital literacy, particularly those concerning critical thinking and information literacy, influenced their readiness for web-based learning, particularly concerning critical thinking and information literacy [31].

Middle-aged individuals tend to trust information from authoritative and professional sources, often considering the information provider’s professional background. This finding is consistent with the results of a previous study [32]. Other research has also shown that middle-aged adults often rely on general practitioners for health information and that health volunteers play a significant role in enhancing their health literacy [32,33]. In contrast, older adults judge an information provider based on factors, such as accessibility, trust, and familiarity with the provider through prior offline consultation support. Some older adults prefer offline means of obtaining information, such as face-to-face medical consultations, due to the personal interactions that they enable and the relationships that they help to establish with health care providers.

Older individuals generally use social media less frequently than younger and middle-aged people to express their emotions. Prior research has also indicated that social media use varies by age [34]. Younger individuals often indirectly express their emotions on social media, preferring to communicate to smaller circles of connections to avoid negative content from being publicly shared [35]. This behavior may be a result of their concern for personal privacy and maintaining a positive image. However, a study of Nigerian adolescents revealed that cultural and socioeconomic factors influenced their expression and control of anger on social media [36], emphasizing the need to foster digital literacy, emotional intelligence, empathy, and respectful digital interactions.

Middle-aged adults usually avoid expressing negative emotions publicly on social media as doing so may lead to personal or professional repercussions. They are more likely to seek support and resolve issues privately instead of publicizing personal emotional struggles, which could affect their family or work. The factors that influence their choices in this regard include privacy concerns, maturity, and the need for support [37,38]. Research has shown that middle-aged and older adults may have more resources and skills to regulate emotions than younger adults, making them less likely to publicly express negative emotions [39].

Older adults rarely use social media to express emotions, primarily due to unfamiliarity with the technology. The “sociodigital divide” within families can result in the social exclusion of older members who are unfamiliar with newer technologies [40], straining intergenerational communication and leading to feelings of alienation. Older adults’ hesitation to engage in typical social media practices, such as chatting and sharing selfies, is often because of a perceived mismatch between social media cultures and the values, interests, or needs of older individuals. This mismatch acts as a social barrier to their digital participation and inclusion [41].

### Implication of the Study and Future Research Direction

To the best of our knowledge, this is the first study spanning multiple age groups to investigate eMHL and the various mechanisms that people use to manage their mental health problems through digital resources. Numerous studies have investigated the eHealth literacy of young people. However, this study focuses on the perspectives of middle-aged and older individuals with mental health issues, comparing them to those of young individuals. It explores their knowledge, skills, and ability to manage these issues, identifying areas where the current mental health system could improve to better serve them. Additionally, these findings are important for improving access to clinical mental health care. They provide empirical evidence that can help clinical professionals design effective interventions to address mental health issues across various age groups. In the future, a quantitative study should be conducted to understand the relationships between eMHL and various socioeconomic factors across different age groups.

### Limitations

This study has some limitations that future work should address. The main limitation of the study is the use of the self-reported DASS-21 to recruit individuals with mental health issues. Although this is a valid instrument to assess mental health, our recruitment process may not have been as reliable as we did not obtain a formal confirmation of the participants’ mental health status via a proof of clinical diagnosis. Another limitation of the study is that we did not interview individuals without mental health problems, who may have been able to provide different perspectives on managing mental health issues.

### Conclusions

This study explored differences in eMHL between 3 age groups in the Chinese population. We identified differences in knowledge, skills, and attitudes regarding the use of digital information to manage mental health problems between young, middle-aged, and older individuals. Younger individuals tend to use a range of digital platforms to manage stress or anxiety, whereas middle-aged individuals tend to rely on information from a few reliable digital sources. In contrast, older adults generally prefer offline consultations with the health care providers that they find via the internet. Our findings highlight the need to develop age group–specific strategies to enhance eMHL. This tailored approach could improve individuals’ ability to identify and access reliable digital resources, thus promoting the effective management of their mental health.

## Abbreviations

eMHL: electronic mental health literacy
DASS-21: Depression, Anxiety and Stress Scale-21 Item

## Data Availability

The data sets generated and analyzed during this study are available from the corresponding author on reasonable request.
